# A Conversation with Sumit Chanda, Antiviral Drug Discovery Scientist

**DOI:** 10.1021/acscentsci.5c02070

**Published:** 2025-11-07

**Authors:** Elizabeth Walsh

## Abstract

Sumit Chanda’s search for antivirals is in limbo after NIH halted its pandemic-preparedness program.

## Graphical abstract

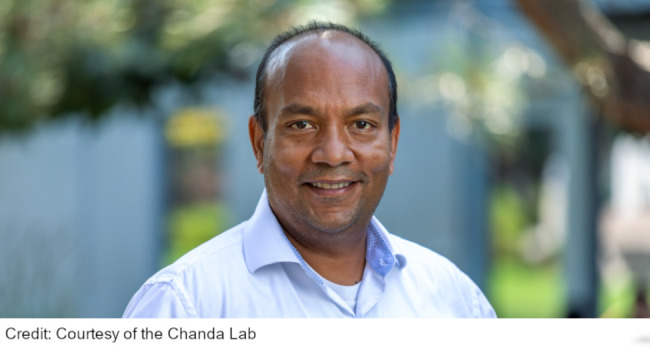

S umit Chanda has built a career around tackling the planet’s most devastating viruses.

After earning his Ph.D. from Stanford University, Chanda developed ways to apply high-throughput screening and systems biology approaches to identify druggable host–virus interactions. He spent years leading drug discovery efforts for antivirals to treat HIV, influenza, and coronaviruses. When COVID-19 hit, he paused everything to screen 12,000 compounds against SARS-CoV-2. He’s now a professor in the Department of Immunology and Microbiology at the Scripps Research Institute and continuing his research on viruses.

In 2022, the US National Institutes of Health (NIH) awarded Chanda an Antiviral Drug Discovery (AViDD) grant that provided him with $67 million to develop drugs against future pandemics and do what he loves most: make antivirals to help people.

**Figure:**
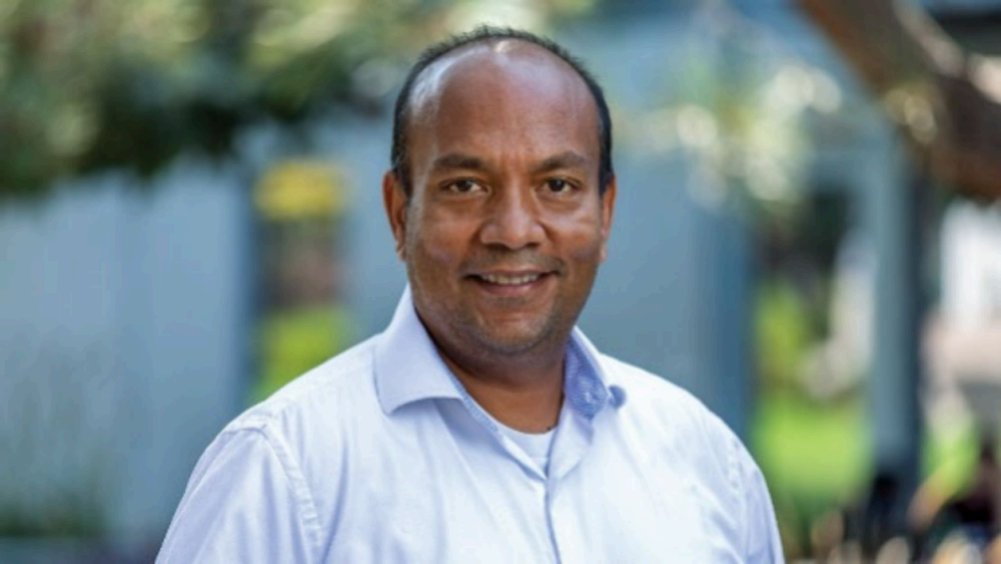
Sumit Chanda has dedicated his career to antiviral drug research. Credit: Courtesy of the Chanda Lab.

But 3 years into the grant, on March 24, 2025, Chanda received an email that said, “As of 5 p.m. today your grant is terminated,” he says. The email arrived at 12:52 p.m.

“It was kind of surreal...It was basically verbiage that said, ‘Well, now that the pandemic’s over, we don’t need to work on this anymore,’” Chanda says.

Since then, he has paused projects and laid off staff. If funding isn’t restored soon, Chanda says, promising antivirals will be lost, and the world will be defenseless against the next pandemic.

## What was AViDD?

Antivirals are an important part of the pharmaceutical arsenal, especially at the early stages of a viral outbreak, Chanda says. That’s because they can start working immediately to reduce contagion and severe disease while vaccines are still in development.

Modeling analysis shows that if just 20% of symptomatic individuals had received early antiviral treatment, over 58% of COVID-19 deaths across several countries, including the US, could have been averted (*BMC Infect. Dis*. 2022, DOI: 10.1186/s12879-022-07639-1). But there are no approved antivirals for four of the eight virus families the NIH identified in 2023 as having the potential to cause future pandemics.

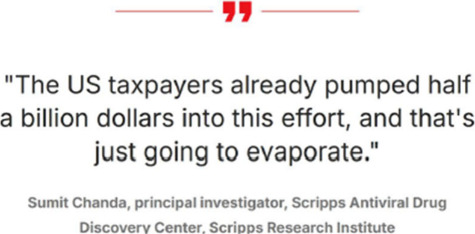



That gap was why the AViDD program was launched. In 2022, the NIH used COVID-19 relief funds to create nine AViDD centers in the US, each charged with developing the next generation of drugs against major viral classes that could cause a pandemic. Chanda’s lab was among them.

Paired with an industry partner to move drugs to clinical trials, each center had 3 years to spend the funds; at the time, the NIH anticipated a 2-year extension and an additional $600 million. But in late 2024, the NIH announced that the 2-year renewal was not happening and instead gave researchers the option of a 1-year, no-cost extension—a standard procedure that lets the team spend funds beyond the end of a grant.

Although it wasn’t what Chanda had hoped for, it would have given him time to secure additional funds, reorganize staff, and complete current projects. But then in March, Chanda and every other AViDD director received notice that the extension was terminated and that funds would be rescinded immediately.

In an email response to C&EN about the termination, an NIH spokesperson says it is focusing on the “chronic disease epidemic” and is reviewing all grants to ensure that they address it. The NIH did not respond to further inquiries about AViDD.

Chanda believes the grant wasn’t looked at closely when funding cuts were being made. “I think they did a keyword search and said, ‘Ah, this is under the COVID relief act.’ No one read that it was for pandemic preparedness,” he says.

## The drugs that are being lost

The pipeline for antiviral drug development follows a standard route: Researchers identify and validate viral targets, find chemical hits, and optimize the lead candidate. After a strong drug is developed, it undergoes preclinical screening, clinical trials, and—if all goes well—regulatory review. According to PhRMA (Pharmaceutical Research and Manufacturers of America), developing a new medicine from initial discovery to regulatory approval takes an average of 10–15 years.

Chanda had projects at nearly every preclinical stage that he says will need to be put on hold or permanently shelved if another funding source doesn’t come through.

One of the antiviral candidates, modified from the antiflu medication baloxavir marboxil, targets Lassa fever virus, which is endemic to West Africa and can cause hemorrhagic fever and death. In 2016, the World Health Organization listed it as a potential pandemic threat that needs urgent research and development.

**Figure:**
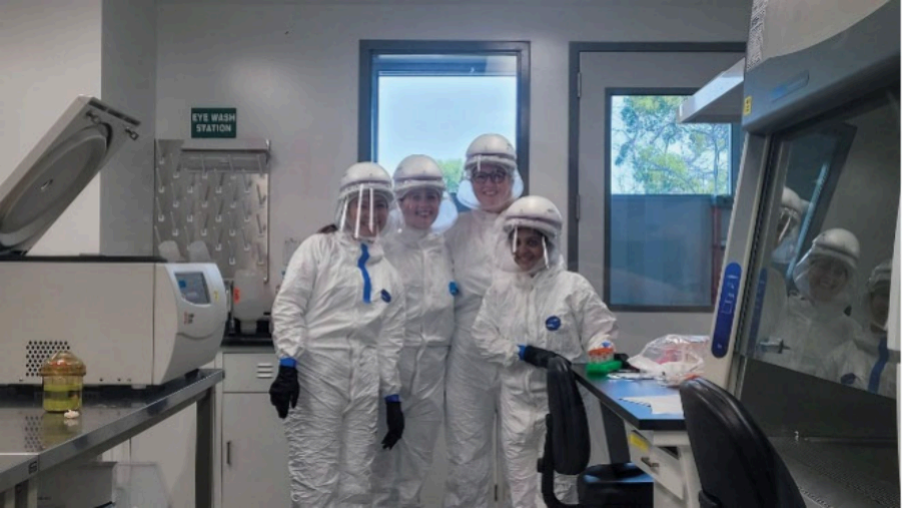
Members of Sumit Chanda’s lab have researched a variety of virus families to find new antivirals. Credit: Courtesy of the Chanda Lab.

“It was like the last experiment that we could fund. It works in mice; it’s safe, it’s effective.... It’s essentially about as good as a preclinical data package as you can get for a treatment for Lassa fever,” Chanda says. “If we had a couple more years, we could’ve scratched this one off.”

Another project now on hold is a broad-spectrum antiviral for treating multiple RNA viruses. Chanda says that though it is still in the early research phases, the candidate showed “extremely” promising data and appeared more comprehensive than remdesivir, a broad-spectrum RNA virus antiviral.

“There’s nothing else I’ve ever seen that looks like this,” Chanda says. “We could cross off 90% of [viral] threats.”

The Scripps AViDD Center isn’t alone; other promising projects across US centers are also in limbo.

A pan-coronavirus antiviral developed by the Artificial intelligence-driven Structure-enabled Antiviral Platform (ASAP) AViDD Center had just completed preclinical toxicology studies when the funding was revoked, says Ed Griffen, the head of lead optimization at ASAP. On September 23, 2025, the Drugs for Neglected Diseases initiative (DNDi), one of the partners in the ASAP consortium, announced that it was nominating ASAP-0017445 as a preclinical drug candidate and calling for donors to support its clinical development.

Multiple other ASAP campaigns will need to be shelved if the center doesn’t secure new funding, Griffen says, including coronavirus projects, a compound aimed at mosquito-borne pathogens, and a lead against enteroviruses.

Rueben Harris, coprincipal investigator at the Midwest AViDD Center, says that when the funding cuts came, the center had an “amazing number” of novel molecules in development focused on targeting essential proteins in SARS-CoV-2, Ebola, Lassa, and Zika viruses. Some of those were being optimized for preclinical development, and while some campaigns have enough data to be published, others might “not see the light of day,” Harris says.

## Pharmaceutical companies not likely to step up

With widespread funding cuts to drug development at the academic level, it has been hard to find new funding sources. Chanda says he does not expect the pharma industry alone to fill the gaps, as there’s no financial incentive until a pandemic occurs.

A 2023 report from the Government Accountability Office (GAO) on antivirals and economic incentives and strategies for pandemic preparedness found that market forces alone are unlikely to induce antiviral drug development at levels that would benefit society.

Publicly traded companies are accountable to shareholders and must demonstrate that a drug will make money, says Steinar Sønsteby, CEO of Atea, a pharmaceutical company focused on antiviral development. “Antivirals are not a priority unless there’s a pandemic.”

But even if there is another pandemic, pharmaceutical firms are disincentivized from developing new antivirals, according to Michael Hoffman, director at the GAO’s Center for Economics. After the COVID-19 crisis, he says, companies know that “they won’t be able to charge a monopoly price for their pandemic antivirals.”

In recent years, several pharmaceutical groups have pulled out of antiviral development. In October 2024, Johnson & Johnson discontinued the Phase 2 study of its dengue antiviral as part of a “strategic reprioritization.” The move followed the discontinuation of seven of the company’s infectious disease programs, and J&J joined other drugmakers that have scaled back infectious disease programs in the past decade.

Griffen says other known diseases with a large patient population have a more-guaranteed return. “With the same investment you could make, why not go and work on oncology...where you can start getting a return on investment.”

Even when industry is involved, pharmaceutical companies don’t often identify viral targets. Instead, they focus on later stages of the process. A STAT10 report found that from 2020 to 2024, around 50% of all drugs approved by the Food and Drug Administration came from academic institutions. By contrast, a 2000 report found that corporate sponsors supplied roughly 75% of US clinical trial funding—one of the final stages of drug development.

## The cost of cutting short

Without new funding sources, AViDD centers have also had to downsize their teams.

“It’s one thing to set aside or pause the development of a bunch of knowledge, which we still know the structures of,” Harris says. “It is pausing the development of careers that may never redevelop. Yeah, that’s not acceptable.”

At Scripps, Chanda had to fire around 40% of his staff because the abrupt cut didn’t give him time to move people or apply for new funding.

Many of these people were on visas and had to return to their home countries, Chanda says. “We lost talent that would have made America a better place.”

And Griffen says things may get worse. “If we don’t get more money, then we will stop everything.” He argues that in the grand scheme, it’s not drug discovery but drug development that’s expensive. “You can run our whole [ASAP] program at a cost less than one executive jet.”

Chanda says that cutting the program now is also a waste of money. “The US taxpayers already pumped half a billion dollars into this effort, and that’s just going to evaporate.”

But predicting pandemics and how to spend money to avoid them isn’t a clear-cut process. “This is a difficult exercise,” Hoffman says. “Congress and [the Department of Health and Human Services Department], for example, are trying to set priorities about future risk that might cut across many different viral families, different types of pandemics, and different degrees of severity.”

For now, Chanda continues research on viruses and antivirals at his Scripps labs and is searching for other funding to pay for development of some of the drugs on hold.

But he worries that the recent funding cuts have made the US less prepared for a future pandemic—and it’s only a matter of time. “Bird flu is the slow-moving train wreck that keeps me up at night,” Chanda says.

He hopes the NIH will reverse the decision and continue the AViDD program.

“This is something that should be transcending any political differences that we have,” Chanda says. “[Viruses] are not resting because we have pandemic fatigue.”

*Elizabeth Walsh is the 2025 Editorial Fellow at* Chemical & Engineering News, *the independent news publication of the American Chemical Society.* A version of this story appeared in C&EN.

